# A novel role for the mono-ADP-ribosyltransferase PARP14/ARTD8 in promoting homologous recombination and protecting against replication stress

**DOI:** 10.1093/nar/gkv147

**Published:** 2015-03-09

**Authors:** Claudia M. Nicolae, Erin R. Aho, Katherine N. Choe, Daniel Constantin, He-Juan Hu, Deokjae Lee, Kyungjae Myung, George-Lucian Moldovan

**Affiliations:** 1 Department of Biochemistry and Molecular Biology, The Pennsylvania State University College of Medicine, 500 University Drive, Hershey, PA 17033, USA; 2 Suzhou Health College, Suzhou, Jiangsu 215009, P.R. China; 3 Genome Instability Section, National Human Genome Research Institute, Bethesda, MD 20892, USA

## Abstract

Genomic instability, a major hallmark of cancer cells, is caused by incorrect or ineffective DNA repair. Many DNA repair mechanisms cooperate in cells to fight DNA damage, and are generally regulated by post-translational modification of key factors. Poly-ADP-ribosylation, catalyzed by PARP1, is a post-translational modification playing a prominent role in DNA repair, but much less is known about mono-ADP-ribosylation. Here we report that mono-ADP-ribosylation plays an important role in homologous recombination DNA repair, a mechanism essential for replication fork stability and double strand break repair. We show that the mono-ADP-ribosyltransferase PARP14 interacts with the DNA replication machinery component PCNA and promotes replication of DNA lesions and common fragile sites. PARP14 depletion results in reduced homologous recombination, persistent RAD51 foci, hypersensitivity to DNA damaging agents and accumulation of DNA strand breaks. Our work uncovered PARP14 as a novel factor required for mitigating replication stress and promoting genomic stability.

## INTRODUCTION

DNA repair mechanisms protect the genomic information against alterations and thus counteract transformation and tumorigenesis (1–3). In particular, homologous recombination (HR) DNA repair is essential for genomic stability and protection against cancer (4–7). Inherited mutations in HR genes result in increased susceptibility to breast, ovarian and other cancers, and somatic mutations are frequently found in sporadic cancers. HR repairs DNA strand breaks employing a generally error-free mechanism, by using the sister chromatid as a template. Cells from patients with mutations in HR genes show increased genomic instability and accumulation of mutations, since in recombination-deficient cells, other, more error-prone repair mechanisms become prominent. On the other hand, HR-mediated DNA repair is a major response of cancer cells against genotoxic chemotherapy. HR-proficient cells show increased resistance to chemotherapy and HR inhibitors have been proposed in cancer therapy as chemosensitizers (8,9). Finally, HR has recently gained recognition in novel personalized therapy approaches for cancer treatment, taking advantage of synthetic lethality interactions between HR and other DNA repair pathways (7,10,11).

During DNA replication, unrepaired DNA lesions or difficult to replicate templates such as those found at common fragile sites (CFS) result in replication arrest. Prolonged stalling of the replication machinery at these lesions can lead to collapse of the replication fork, and double strand break formation (1,12–15). This is a major cause for genomic instability in both normal and cancer cells, and it is believed to represent a major mechanism of carcinogenesis, by allowing cells to accumulate mutations and acquire cancer phenotypes (16–18).

Two major mechanisms are available to cells for restarting stalled replication forks: HR and translesion synthesis (TLS) (1,4,12,13,19). HR can be initiated at stalled forks to re-establish replication following formation of a recombination structure called displacement (D) loop. Essential to HR is the protein RAD51, which is loaded by BRCA2 on the DNA end and catalyzes D-loop formation (5,20). In contrast, TLS employs specialized low-fidelity polymerases, able to replicate through difficult templates, including DNA lesions (21,22). These polymerases frequently introduce mutations and represent a major mechanism for point mutagenesis in human cells. Because of their different mechanisms and outcomes, cells tightly regulate replication restart pathways. We and others previously showed that a major regulatory mechanism is represented by post-translational modifications of the replication fork component PCNA, a homo-trimeric ring-shaped protein that encircles DNA and provides processivity to DNA polymerases (23–29). PCNA mono-ubiquitination recruits TLS polymerases through their tandem PCNA-interacting (PIP) and ubiquitin-interacting (UIM) motifs. In contrast, PCNA SUMOylation recruits proteins that block HR by antagonizing with RAD51.

ADP-ribosylation is a prominent post-translational modification that functions in many cellular processes including regulation of transcription and signal transduction (30–34). PARP1 (ARTD1), the founding member of the ADP-ribosyltransferase family (also known as poly-ADP-ribose polymerases, or PARPs) catalyzes poly-ADP-ribose (PAR) chain formation on various substrates including itself. PARP1 participates in many cellular processes including DNA repair, through regulation of base excision repair and signaling at double strand breaks. Unlike PARP1, a subset of the PARP family members cannot catalyze PAR chain formation, but can only transfer a single ADP-ribose molecule. The roles of these mono-ADP-ribosyl (MAR)-tranferases, including PARP10 (ARTD10) and PARP14 (ARTD8, BAL2), are much less understood, and functions of MARylation in DNA repair are only now being uncovered. We recently showed that PARP10 contains PIP and UIM domains which recognized ubiquitinated PCNA (35). We found that PARP10 collaborates with ubiquitinated PCNA to promote error-prone TLS and mutagenesis in human cells. PARP10 is essential for resistance to replication fork stalling agents such as hydroxyurea (HU) and ultraviolet (UV) light, and promotes recruitment of TLS polymerases to sites of replication fork stalling.

PARP14 is structurally and functionally related to PARP10. The two proteins share substrates, including GSK3β and histones, and participate in common pathways including regulation of NFkB activity (36,37). However, PARP14 has also been described as a transcriptional regulator in B-cells (38), and to localize to focal adhesions and participate in cell attachment in HeLa cells (39). PARP14 contains three specialized macrodomains, which can interact with ADP-ribosylated proteins (30,40). In particular, the Macro2 domain of PARP14 can specifically recognize mono-ADP-ribosylation, such as that catalyzed by PARP10. This makes PARP14 a unique protein that is both modifier, through its PARP domain-mediated MARylation activity, and reader of this modification, through its Macro2 domain. Here, we investigate the role of PARP14 in DNA repair, and we show that PARP14 is essential for genomic stability by promoting HR and alleviating replication stress.

## MATERIALS AND METHODS

### Cell culture and protein techniques

Human HeLa, 293T, 8988T and U2OS cells were grown in Dulbecco's modified Eagle's medium (Lonza) supplemented with 15% fetal calf serum. Co-immunoprecipitation and GST-pulldown experiments (41), chromatin fractionation (42) and immunofluorescence (35,43) were done as described. Antibodies used were: PCNA, H2AX and γH2AX (Abcam), PARP14, RAD51 and Actin (Santa Cruz Biotechnology), control IgG (Genescript) and Flag (Sigma). For immunoprecipitation experiments, RAD51 NA-71 antibody (Calbiochem) was used. Quantitative reverse transcriptase-polymerase chain reaction (RT-PCR) was previously described (43). The PARP14 primers used were AGACACAAAGGGCCACAG (forward) and CCAGAGATCTGGATTCTG (reverse).

### Plasmids and small interfering RNA

The cDNA for PARP14 Macro2 was synthesized by Invitrogen Gene-Art Synthesis. Plasmid and small interfering RNA (siRNA) transfections were performed using Lipofectamine LTX and Lipofectamine RNAiMAX (Invitrogen). For gene knockdown, cells were transfected with siRNA twice, in consecutive days, and analyzed 72 h after the first transfection. The siRNA sequences used are shown below (if the oligo number is not shown in the figure, the sequence #1 was employed). As controls, AllStars Negative Control (Qiagen) and Stealth RNAi Negative Controls (Invitrogen) were employed.

siPARP14 #1: AGGCCGACTGTGACCAGATAGTGAA;

siPARP14 #2: CGGCACTACACAGTGAACTTGAACA;

siPARP10: GCCTGGTGGAGATGGTGCTATTGAT.

### Functional assays

Cell cycle, BrdU incorporation assays and clonogenic assays were previously described (27,35,43). SupF assays for measuring mutagenesis frequency (44) and HR assays using the DR-GFP reporter (45) were performed as previously described. For alkaline comet assay, the CometAsssay Kit (Trevigen) was used, according to manufacturer's instructions. For statistical analyses, the *P*-values shown in graphs (*** indicating *P* < 0.001; ** indicating 0.001 < *P* < 0.01; * indicating 0.01 < *P* < 0.05) were calculated using the T-Test (two-tailed distribution, two-sample unequal variance).

### DNA fiber assay

Cells were incubated with 20-μM CldU for 30 min, then media was washed and fresh media containing 20-μM IdU and 1-μM CPT was added for 30 min. Cells were lysed in 0.5% sodium dodecyl sulphate (SDS), 200-mM Tris-HCl pH 7.4, 50-mM ethylenediaminetetraacetic acid (EDTA), and chromatin fibers were stretched on glass slides. Slides were fixed with methanol:acetic acid (3:1) solution for 5 min, washed with 2.4N HCl and blocked in 5% bovine serum albumin in phosphate buffered saline (PBS). Slides were then incubated with primary antibodies (Abcam 6326-detects CldU; BD 347580-detects IdU), and subsequently with secondary Alexa antibodies (Invitrogen A21208 and Invitrogen A11031).

### BrdU/propidium iodide double staining assay

Two-dimensional BrdU/propidium iodide (PI) flow cytometry was performed as previously described (46). Briefly, cells were incubated with 20-μM BrdU for 30 min, then harvested by trypsinization and fixed in 70% ethanol overnight. Cells were incubated in 0.1-M HCl/0.5% Triton X- 100 in PBS for 10 min on ice. Samples were spun down, resuspended in water and boiled for 10 min and then placed on ice for 10 min. Samples were then incubated with primary antibody (Anti-BrdU, BD 347580) and subsequently with secondary AlexaFluor antibody (Invitrogen A11013) for 30 min each. Before flow cytometry analysis, cells were resuspended in PBS containing 20-μg/ml RNase and 5-μg/ml PI.

### Chromatin immunoprecipitation assay

Cells were crosslinked with formaldehyde (1% final concentration), quenched with glycine (125-mM final concentration) and lysed in 1% SDS, 10-mM EDTA, 50-mM Tris-HCl pH 8. Following sonication, the lysate was diluted 10-fold in Tris-HCl pH 8 buffer, and Triton X100 was added to 1% final concentration. Extracts were incubated overnight with 5 μg of PARP14 or control mouse IgG, and Protein A/G Sepharose (Santa Cruz Biotechnology). The beads were washed in 10-fold diluted lysis buffer and eluted in 1% SDS, 0.1-M NaHCO_3_. Eluates were treated with RNase and Proteinase K, and DNA was purified with a PCR purification kit (Omega). Resulted DNA was dissolved in water and subjected to qRT-PCR using FRA3B (for: ACGTGTGAATGAGGCAGAGA; rev: GTTTGTGGGGTGCTATGCTT), FRA7H (for: TAATGCGTCCCCTTGTGACT; rev: GGCAGATTTTAGTCCCTCAGC) and GAPDH (for: TGCACCACCAACTGCTTAGC; rev: TCAGCTCAGGGATGACCTTG) (47–49).

## RESULTS

### PARP14 associates with PCNA during S-phase

**Figure 1.**
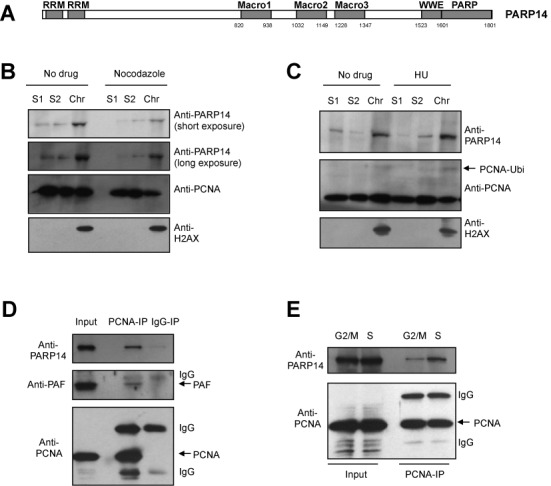
PARP14 associates with chromatin and PCNA in S-phase. (**A**) Schematic representation of PARP14. Macro2 is the mono-ADP-ribose binding domain, while PARP is the mono-ADP-robosyltransferase catalytic domain. (**B, C**) Chromatin fractionation experiments in HeLa cells, showing that PARP14 binding to chromatin is reduced in G2/M (samples treated with 100-ng/ml nocodazole for 18 h) and increased following replication arrest (samples treated with 2-mM HU for 24 h). S1 and S2 represent soluble fractions, while Chr indicates the chromatin fraction. H2AX is used as control for the chromatin fraction. (**D**) Co-immunoprecipitation experiment showing that endogenous PCNA and PARP14 interact in 8988T cells. The PARP14–PCNA interaction is unlikely to be mediated by DNA, since addition of benzonase does not reduce it (Supplementary Figure S1C). (**E**) The PCNA immunoprecipitation experiment was repeated from 8988T cells arrested in S-phase (by 2.5-mM thymidine treatment for 18 h, followed by recovery for 2 h in drug-free media), or G2/M (by 100-ng/ml nocodazole treatment for 18 h). PARP14 co-precipitated with PCNA preferentially in S-phase.

We previously showed that the mono-ADP-ribosyltransferase PARP10/ARTD10 is required for DNA damage tolerance and interacts with PCNA to promote TLS (35). PARP14 is structurally related to PARP10, sharing RRM domains in their N-termini and mono-ADP-ribosylation domains in their C-termini (Figure 1A). We wondered whether PARP14 might also be involved in DNA replication fork stability. Using chromatin fractionation assays, we found that PARP14 is predominantly found bound to chromatin (Figure 1B). When we treated cells with nocodazole to arrest them in G2/M, the amount of chromatin-bound PARP14 decreased; a similar decrease was observed for the replication fork component PCNA. In contrast, the amount of chromatin-bound PARP14 increased when cells were treated with the replication fork stalling agent HU, which induces PCNA ubiquitination (Figure 1C). These results prompted us to investigate if PARP14 is physically connected to the DNA replication machinery. Using co-immunoprecipitation experiments with endogenous proteins in 8988T cells, we found that PARP14 interacts with PCNA (Figure 1D). The previously described PCNA interaction with PAF (50) is used as positive control. The PCNA–PARP14 interaction was detected predominantly in cells that were synchronized in S-phase, and much less in G2/M-arrested cells (Figure 1E). Similar results were obtained when using HeLa cells (Supplementary Figure S1). Moreover, we found that recombinant GST-tagged PCNA was able to interact with PARP14 from native extracts of HeLa cells (Supplementary Figure S2). These results show that PARP14 is associated with the DNA replication machinery component PCNA.

### PARP14-depleted cells show hypersensitivity to replication stress

**Figure 2.**
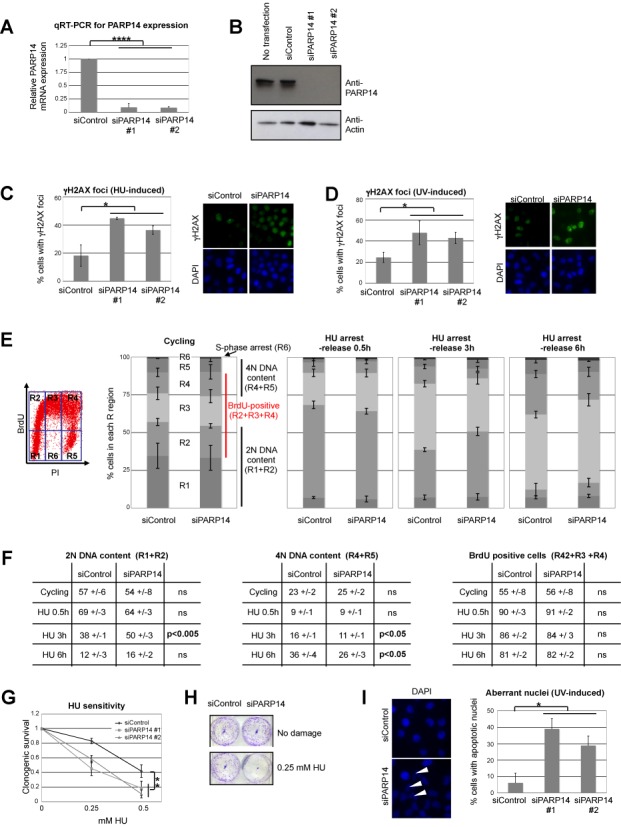
PARP14 knockdown sensitizes cells to replication fork stalling agents. (**A**) Quantitative RT-PCR experiments, showing that PARP14 siRNA oligonucleotides efficiently downregulate PARP14 mRNA in HeLa cells. Bars represent average of three experiments, error bars are standard deviations. (**B**) Western blot with anti-PARP14 antibody showing that PARP14 protein levels are also efficiently downregulated by siRNA in HeLa cells. (**C, D**) Representative micrographs and quantification of the percentage of HeLa cells with strong γH2AX staining following exposure to HU (1 mM for 12 h) or UV (20 J/m^2^, analyzed 2 h later). The average of three independent experiments is shown, error bars representing standard errors. No significant difference between control and PARP14-depleted cells was observed in the absence of DNA damage (Supplementary Figure S4A). (**E**) BrdU/PI double staining and bi-dimensional flow cytometry were performed in control, or PARP14-knockdown HeLa cells. Cycling as well as HU-arrest (2 mM for 18 h)/release samples were analyzed as indicated. An example of flow cytometry plot is shown, indicating the regions that were quantified: R1 = G1 cells (BrdU-negative, 2N DNA content); R2 = early S-phase cells (BrdU-positive, 2N DNA content); R3 = mid-S-phase cells (BrdU-positive, DNA content between 2N and 4N); R4 = late S-phase cells (BrdU-positive, 4N DNA content); R5 = G2 cells (BrdU-negative, 4N DNA content); R6 = S-phase-arrested cells (BrdU-negative, DNA content between 2N and 4N). The graphs show the quantification of R1-R6 regions, averaged from three independent experiments, with standard deviations indicated as error bars. Representative flow plots are shown in Supplementary Figure S5. (**F**) The percentage of 2N DNA containing cells (R1+R2), 4N DNA containing cells (R4+R5) and of BrdU positive cells (R2+R3+R4) is shown (averaged from three independent experiments, ± standard deviations). *P*-values are also shown (ns = not significant). (**G**) Clonogenic survival assay showing that PARP14-knockdown HeLa cells are sensitive to HU treatment. Cells were treated with indicated doses of HU for 48 h, then incubated in fresh, drug-free media for 2 weeks. The average of three independent experiments, with standard deviations as error bars, is shown. Similar results were obtained in 8988T cells (Supplementary Figure S7). (**H**) Representative clonogenic assay showing HU sensitivity of PARP14-depleted HeLa cells. (**I**) UV (200 J/m^2^) -induced loss of viability is increased in the absence of PARP14. Left: representative micrographs showing accumulation of cells with aberrant multi-lobed nuclei (pre-apoptotic cells) 2 h after UV irradiation. HeLa cells were analyzed by DAPI staining. Right: quantification of at least three experiments. Shown are averages, and error bars represent standard errors. At least 25 cells were counted.

We next investigated how PARP14 depletion impacts the response to DNA damage. Two different siRNA oligonucleotides were able to efficiently reduce the levels of PARP14 in HeLa, U2OS and 8988T cells (Figure 2A and B and Supplementary Figure S3). PARP14 knockdown in HeLa cells resulted in increased γH2AX staining following exposure to replication fork stalling agents HU and UV (Figure 2C and D), while no difference was observed in the absence of drug treatment (Supplementary Figure S4A). Similar results were obtained in U2OS cells (Supplementary Figure S4B and C). These results suggest an inability to process stalled replication forks in the absence of PARP14. We next monitored cell cycle distribution in control and PARP14-knockdown HeLa cells. We treated HeLa cells with BrdU and performed anti-BrdU and PI double staining followed by bi-dimensional flow cytometry (Figure 2E and F and Supplementary Figure S5). The percentage of BrdU-positive cells and the general cell cycle distribution were not affected by PARP14 knockdown in the absence of drug treatment. We next treated cells with 2-mM HU for 18h and analyzed them at several time points following drug removal. PARP14 knockdown cells showed a reduced percentage of cells in mid-S-phase 3 hours after release, and a reduced percentage of cells in late S-phase 6 hours after release (Figure 2E and Supplementary Figure S5). The percentage of cells with replicated DNA (4N DNA content) was significantly reduced in PARP14 knockdown cells 3 and 6 h after drug removal (Figure 2F and Supplementary Figure S6). These results further indicate that PARP14 knockdown cells have a significant defect in progression through S-phase under replication stress conditions. Indeed, we found that PARP14-depleted cells were sensitive to HU in clonogenic survival assays (Figure 2G and H and Supplementary Figure S7). Moreover, we observed an increase in aberrant, multi-lobed pre-apoptotic nuclei in PARP14 knockdown HeLa cells after UV exposure, suggesting that PARP14 is important for protecting cells against UV damage (Figure 2I). Altogether, these results show that PARP14 is important for DNA replication under stress conditions.

### Efficient HR requires PARP14

**Figure 3.**
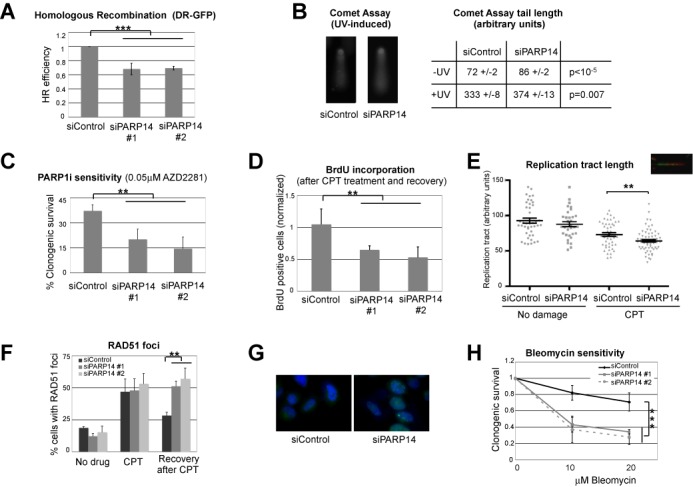
PARP14 knockdown reduces homologous recombination. (**A**) DR-GFP HR assay in U2OS cells. PARP14 knockdown by two different siRNA oligonucleotides reduces HR efficiency. The bars represent the average of three independent experiments, error bars represent standard deviations. (**B**) Increased single and double strand breaks in PARP14-depleted cells as measured by the alkaline comet assay. Representative micrographs of control and PARP14-depleted HeLa cells comets, after UV exposure (40 J/m^2^ analyzed 4 h later), are shown. The average comet tail length from three experiments (± standard errors), with 25–40 comets measured in each assay, from not damaged and UV-exposed cells, is presented in the table (arbitrary units). The *P*-value calculated for all measurements in the three experiments is shown. Similar results were obtained in U2OS and 8988T cells (Supplementary Figure S9). (**C**) Clonogenic experiment showing that PARP14 knockdown HeLa cells are sensitive to PARP1 inhibitor AZD2281. The average of three independent experiments, with standard deviations as error bars, is shown. (**D**) BrdU incorporation experiment showing reduced replication following CPT exposure of HeLa cells with PARP14 siRNA. Cells were treated with 1-μM CPT for 2 h, then released in drug-free media, and 4 h later were incubated with BrdU. The average of three experiments is shown, error bars are standard errors. (**E**) DNA fiber assay showing reduced replication tract length in PARP14 knockdown HeLa cells treated with 1-μM CPT. At least 40 fibers for each condition, from two independent experiments, were measured and included in the analysis. Error bars represent standard errors. Similar results were obtained in U2OS and 8988T cells (Supplementary Figure S10). (**F, G**) RAD51 foci in PARP14-depleted HeLa cells. Shown is the percentage of cells with more than five RAD51 foci under basal conditions, upon treatment with 1-μM CPT for 2 h, and 4 h following drug removal (‘Recovery’). The average of three independent experiments, with standard errors as error bars, is shown. Representative micrograph of the recovery condition is also shown. Similar results were obtained in U2OS cells (Supplementary Figure S11). (**H**) Clonogenic experiment showing that PARP14 knockdown HeLa cells are hypersensitive to the drug bleomycin, which induces double strand breaks. The average of three independent experiments, with standard deviations as error bars, is shown. Similar results were obtained in 8988T cells (Supplementary Figure S12).

Two main mechanisms can restart stalled replication forks in human cells: TLS and HR. In order to tease out which of these mechanisms might require PARP14, we employed established cell-based assays to measure the efficiencies of these pathways in PARP14-depleted cells. Unexpectedly, PARP14 knockdown cells showed reduced HR (Figure 3A) as assayed by the DR-GFP assay (45), and normal levels of TLS (Supplementary Figure S8) as assayed by the SupF UV-induced mutagenesis assay (44). Indeed, using the comet assay, we observed that PARP14-depleted cells have increased DNA breaks—a hallmark of HR deficiency (Figure 3B and Supplementary Figure S9). Another hallmark of HR deficiency is hypersensitivity to inhibition of PARP1, since single strand breaks cannot be repaired when PARP1 is inactivated, and are transformed to double strand breaks when replication forks reach them (10,11). These S-phase-generated double strand breaks require HR for repair and replication completion. PARP14-depleted HeLa cells were also sensitive to PARP1 inhibition (Figure 3C), again showing that HR is deficient in these cells.

Next, we investigated that the cellular response to camptothecin (CPT), a topoisomerase inhibitor that similarly to PARP1 inhibitors, creates replication-dependent double strand breaks. We focused on studying how replication is affected by CPT treatment in PARP14-depleted HeLa cells. We first quantified replicating cells by BrdU incorporation experiments. As shown earlier (Figure 2E and F), PARP14 knockdown cells have normal replication profile. However, they showed significant reduction in BrdU incorporation 4 h after CPT removal (Figure 3D), suggesting that they are unable to repair double strand breaks and restart replication. We next employed the DNA fiber assay to measure the length of the replication tract in the presence of CPT. While both control and PARP14-depleted cells showed reduced replication tracts in the presence of CPT, as expected, the knockdown cells showed a more drastic reduction (Figure 3E and Supplementary Figure S10), confirming a role of PARP14 in promoting replication of damaged DNA. Finally, we investigated foci formation by RAD51, which is frequently used as readout of HR. Control cells and PARP14 knockdown HeLa cells showed similar levels of RAD51 foci, both under basal conditions and upon CPT exposure. However, in contrast to control cells, PARP14-depleted cells showed persistent RAD51 foci 4 h after CPT treatment (Figure 3F and G and Supplementary Figure S11), suggesting that loss of PARP14 renders cells defective in the clearing of CPT-induced DNA damage.

HR participates both in restarting stalled replication forks in S-phase and in double strand break repair in G2. To investigate if PARP14 is also important for G2 HR, we employed clonogenic assays to assess the sensitivity of PARP14-depleted cells to bleomycin, a chemotherapy drug that creates double strand breaks. PARP14 cells were indeed hypersensitive to bleomycin, suggesting a general role for PARP14 in promoting HR (Figure 3H and Supplementary Figure S12).

### Mono-ADP-ribosylation is required for HR

**Figure 4.**
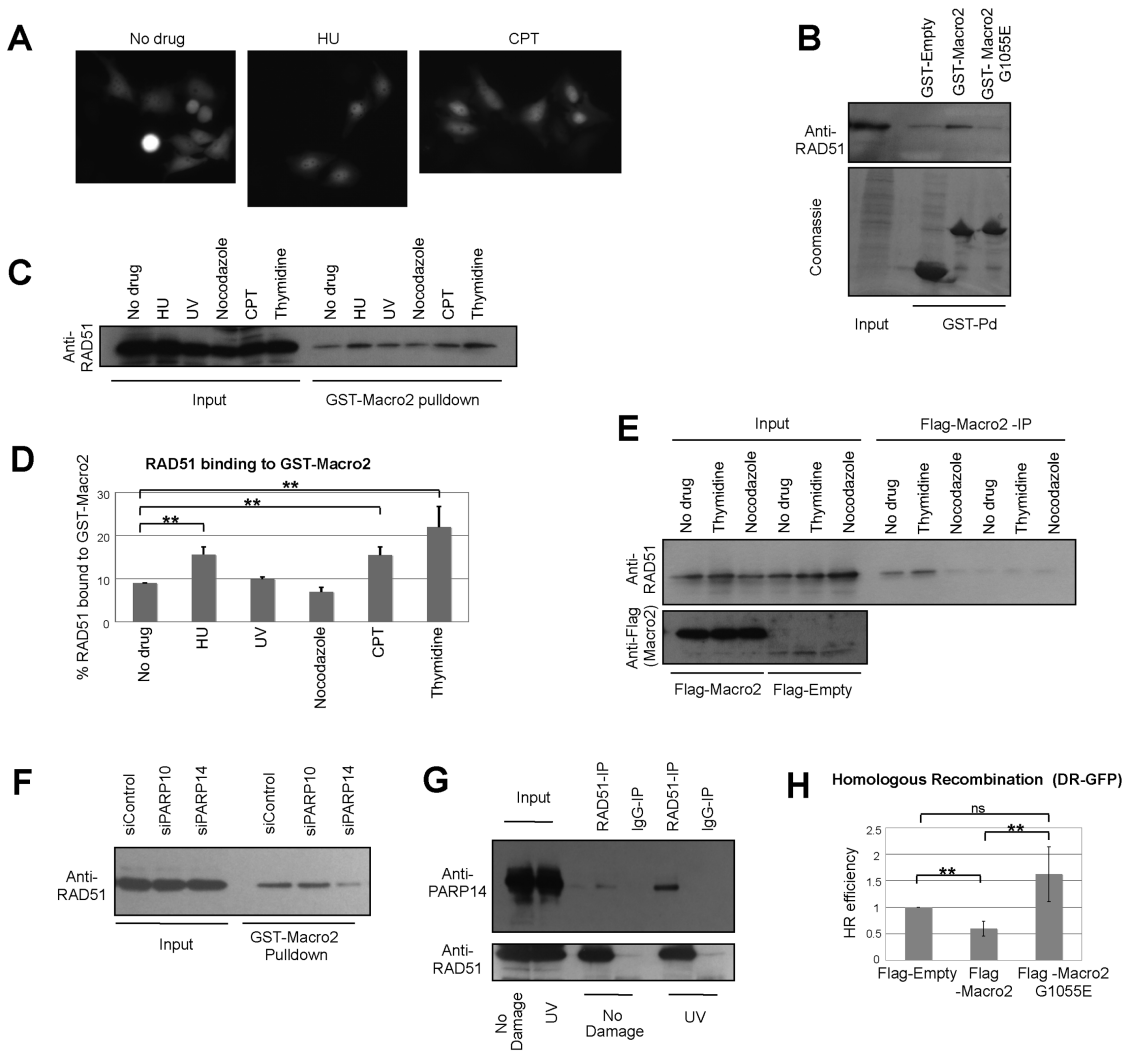
The Macro2 domain of PARP14 and its role in HR. (**A**) GFP-tagged Macro2 overexpressed in HeLa cells localizes throughout the cell under resting conditions, but re-localizes to the nucleus in response to DNA damage (2-mM HU for 18 h and 1-μM CPT for 2h). Representative micrographs of live cell green fluorescence are shown. (**B**) GST-pulldown using recombinant GST-Macro2 or the G1055E mutant. The recombinant proteins were incubated with native extracts of HeLa cells, and binding of endogenous RAD51 was detected by anti-RAD51 antibodies. (**C, D**) GST-pulldowns with recombinant Macro2 and extracts of HeLa cells treated with drugs including HU (1 mM for 18 h), UV (100 J/m^2^ analyzed 2 h after exposure), CPT (1 μM for 2 h), nocodazole (100 ng/ml for 18 h, to arrest cells in G2/M), thymidine (2.5 mM for 18 h, analyzed 2 h after drug removal, to synchronize cells in S-phase). The quantification shows the average of three independent experiments (with standard deviations), analyzed by ImageJ software. RAD51 binding was normalized to the amount in the Input for each condition. (**E**) Flag-tagged Macro2 was overexpressed in HeLa cells and extracts were subjected to Flag-immunoprecipitation. RAD51 binding in cells treated with thymidine or nocodazole (as above) is shown. (**F**) GST-Macro2 pulldowns with extracts of UV-treated (100 J/m^2^ analyzed 2 h after exposure) control, PARP10- and PARP14-depleted HeLa cells. PARP14, but not PARP10 depletion, reduces the Macro2–RAD51 interaction (**G**) Co-immunoprecipitation experiments showing that endogenous PARP14 and RAD51 interact in UV-treated HeLa cells (50 J/m^2^ analyzed 2 h after exposure). (**H**) DR-GFP HR assay in U2OS cells. Flag-tagged Macro2, or the G1055E mutant, was co-expressed with the SceI nuclease. The bars represent the average of four independent experiments, error bars represent standard deviations.

PARP14 is not only a mono-ADP-ribosyltransferase but also a reader of this modification, through its Macro2 domain (40). Because of its ability to bind mono-ADP-ribosylated proteins, we employed the Macro2 domain of PARP14, to investigate how this modification participates in replication fork stability. Using fluorescence microscopy, we found that GFP-tagged Macro2 overexpressed in HeLa cells localizes diffusely throughout the cytoplasm and nucleus. However, after exposure to HU or CPT, GFP-Macro2 relocalized to the nucleus (Figure 4A), suggesting that nuclear proteins became mono-ADP-ribosylated (MARylated) after DNA damage. We next considered the possible identity of these proteins. Since PARP14 participates in HR, and RAD51 localization after DNA damage is affected by PARP14 downregulation (Figure 3), we decided to investigate if RAD51 might be MARylated. We found that recombinant GST-tagged Macro2, but not the variant G1055E, previously shown to lack the MARylation recognition activity, can specifically recognize RAD51 from HeLa cell extracts (Figure 4B), suggesting that RAD51 might be MARylated. To investigate under what conditions this putative MARylation of RAD51 might occur, we repeated this experiment using extracts of HeLa cells that prior to lysis, were treated with DNA damaging agents or drugs that manipulate cell cycle progression. We found a specific increase in RAD51 interaction with Macro2 after treatment with DNA damaging agents including HU and CPT (Figure 4C and D). We also found an increase in this interaction in cells synchronized in S-phase with thymidine, but a decrease in cells synchronized in G2/M with nocodazole. We also observed this pattern in co-immunoprecipitation experiments using flag-tagged Macro2 overexpressed in HeLa cells (Figure 4E). These results suggest that RAD51 becomes MARylated in S-phase and after DNA damage, which are also the conditions in which HR is activated. Importantly, in PARP14 knockdown cells binding of Macro2 to RAD51 is greatly reduced, showing that PARP14 is the primary enzyme responsible for RAD51 MARylation (Figure 4F and Supplementary Figure S13A–C). Indeed, knocking down PARP10, the other major mono-ADP-ribosyltransferase, did not affect the Macro2-RAD51 binding. Moreover, co-immunoprecipitation experiments showed that endogenous RAD51 and PARP14 interact in UV-irradiated cells (Figure 4G and Supplementary Figure S13D). These results argue that PARP14 MARylates RAD51 in response to DNA damage.

To investigate the role of MARylation in HR, we overexpressed flag-tagged Macro2 in U2OS cells and measured HR using the DR-GFP assay. We reasoned that Macro2 should bind to MARylated substrates critical for HR, such as RAD51, and act in a dominant negative fashion by blocking its recognition by downstream binding partners (‘readers’). Indeed, wild-type Macro2, but not the G1055E mutant, resulted in decreased HR (Figure 4H), suggesting that MARylation is important for HR.

### PARP14 binds to CFS and alleviates replication stress

**Figure 5.**
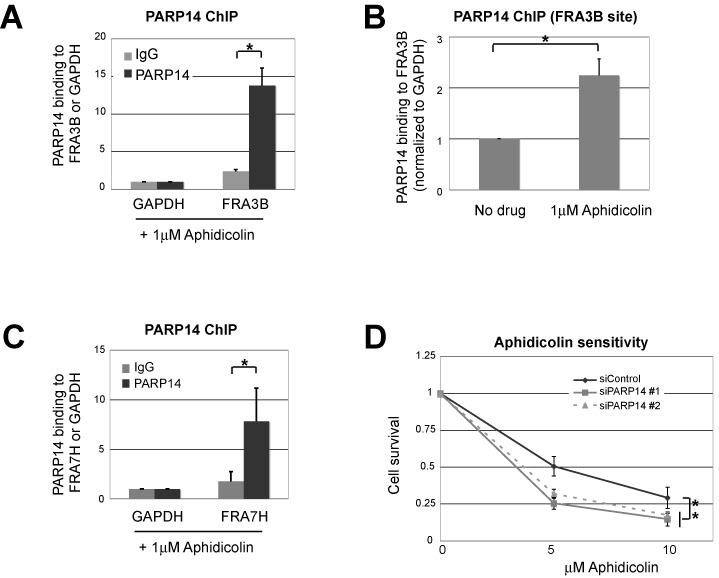
PARP14 localizes to the common fragile site FRA3B and is required for aphidicolin resistance. (**A**) Chromatin immunoprecipitation assay showing that PARP14 is localized to the FRA3B site in HeLa cells. IgG immunoprecipitation was used as control. Samples were treated with aphidicolin (1 μM for 12 h before processing). Chromatin immunoprecipitates were assayed by PCR using primers for the FRA3B common fragile site, and the GAPDH locus as non-CFS control (used for normalization). The average of three independent experiments is shown; error bars are standard errors. (**B**) A similar experiment was performed in HeLa cells in the presence or absence of aphidicolin (1 μM for 12 h). Binding to the GAPDH locus was used for normalization. The average of three independent experiments, with standard deviations as error bars, is shown. (**C**) ChIP assay showing that PARP14 is localized to the FRA7H site in HeLa cells. Samples were treated with aphidicolin (1 μM for 12 h before processing). IgG immunoprecipitation was used as control, and binding to the GAPDH locus was used for normalization. The average of three independent experiments, with standard errors as error bars, is shown. (**D**) Cell survival assay showing that PARP14 knockdown in HeLa cells results in aphidicolin sensitivity. Cells were incubated for 3 days in the presence of indicated aphidicolin doses, and viability was measured using the CellTiterGlo reagent. The average of three independent experiments, with standard deviations as error bars, is shown. Similar results were obtained in 8988T cells (Supplementary Figure S14).

We next wondered if PARP14 is involved in response to endogenous replication stress. CFS are sites in the genome that induce replication fork stalling due to their sequence properties including repetitive elements and the ability to form secondary structures after helicase unwinding, leading to chromosomal breaks and translocations, which can be exacerbated experimentally by treatment with the polymerase inhibitor aphidicolin (14,15). HR has been shown to be required, together with other DNA repair pathways, for replication through CFS and aphidicolin resistance (47,48,51,52). Using chromatin immunoprecipitation (ChIP) assays, we found that PARP14 specifically binds to the FRA3B CFS in HeLa cells, compared to the control non-CFS GAPDH locus (Figure 5A). This binding was increased by aphidicolin treatment (Figure 5B). Moreover, PARP14 also bound to a different CFS, namely FRA7H, in aphidicolin-treated cells (Figure 5C). Finally, we found that PARP14 knockdown renders HeLa cells sensitive to aphidicolin, a hallmark of replication stress (Figure 5D and Supplementary Figure S14). These results suggest that PARP14 is important for suppressing CFS breakage and maintaining genomic stability under normal replication conditions.

## DISCUSSION

### Novel roles for mono-ADP-ribosylation in DNA damage tolerance and repair

The ability to repair DNA damage is fundamental for all cells (1,2,53). Inactivation of DNA repair genes predisposes to cancer by allowing cells to accumulate mutations. On the other hand, cancer cells employ DNA repair mechanism to fight against genotoxic chemotherapy such as radiation, platinum compounds, topoisomerase inhibitors and others. Understanding how cancer cells handle this apparent paradox will allow for more judicious use of genotoxic therapy and DNA repair inhibitors in cancer treatment. While the enzymology and mechanisms of the major DNA repair mechanisms have been extensively characterized, only now we begin to understand how these pathways are regulated to ensure efficient and timely repair.

DNA replication can be a major source of mutations, both due to replication errors, and to the inhibitory effect of DNA damage on replicative DNA polymerases (1,13,16,17). In particular, hyper-proliferating cells may have reduced time available for repairing DNA damage, and many lesions escape repair prior to DNA replication. Unrepaired DNA lesions block the progression of the replication fork and can induce chromosome breaks and translocations. Two major pathways, HR and TLS, can be employed by cells to restart stalled replication forks and bypass DNA lesions. HR is predominantly error free, but more complex and lengthy, while TLS is faster but can introduce mutations. DNA repair is generally regulated by post-translational modifications of essential components of repair pathways (26,53). For example, PCNA modifications by ubiquitin and SUMO promote TLS and block HR, respectively. Poly-ADP-ribosylation catalyzed by PARP1 regulates base excision repair and the signaling at double strand breaks (32). In contrast, very little is known about mono-ADP-ribosylation in DNA repair. We recently showed that the mono-ADP-ribosyltransferase PARP10 promotes PCNA ubiquitination and TLS (35). Here, we report that, surprisingly, the PARP10-related mono-ADP-ribosyltransferase PARP14 is required for HR rather than TLS. Our results suggest that PARP10 and PARP14, although related structurally and functionally, evolved to promote different mechanisms of DNA damage tolerance. Our work brings new light on ADP-ribosylation, highlighting its widespread use in regulating DNA repair well beyond the established PARP1-mediated poly-ADP-ribosylation.

### A novel role for PARP14 in HR

We found that PARP14 knockdown cells are hypersensitive to DNA damaging agents, including those that induce replication fork stalling such as HU and UV and those that induce double strand breaks such as bleomycin and camptothecin (Figures 2 and 3). Since HR is essential for protecting and restarting stalled replication forks and for repair of strand breaks at replication forks, we investigated whether PARP14 is required for HR. Using a well-characterized HR reporter assay, we found that PARP14 depletion impacts HR efficiency (Figure 3). PARP14 knockdown cells correctly arrest their replication after HU and CPT exposure and initiate the formation of RAD51 foci. However, they fail to efficiently restart replication and clear RAD51 foci after the damaging agent was removed, suggesting that repair is initiated, but it is not completed (Figures 2 and 3). Indeed, γH2AX foci and DNA strand breaks accumulate in these cells, showing their inability to clear DNA damage.

The Macro2 domain of PARP14 recognizes mono-ADP-ribosylation on substrates (40). We provide two lines of evidence indicating a role for ADP-ribosylation in HR: first, we found that Macro2 binds to RAD51 in S-phase and following DNA damage (Figure 4), arguing that RAD51 is MARylated under conditions where HR is active. Second, Macro2 overexpression blocked HR (Figure 4), suggesting that the Macro2 domain acts as dominant negative and blocks the HR-promoting activity of the MARylation. These results indicate that post-translational modification of RAD51 by MARylation is required for efficient HR. While we cannot rule out that other enzymes participate in RAD51 MARylation, our data suggest that PARP14 itself might be responsible, since its knockdown reduces both HR and the MARylation-dependent interaction of RAD51 with Macro2—which is not the case for PARP10 knockdown (Figures 3 and 4). It is unclear what the role of RAD51 MARylation is. Our experiments argue that PARP14 is involved in HR at a step following RAD51 foci formation and before RAD51 removal that precedes D-loop extension. Thus, we speculate that PARP14 and RAD51 MARylation might be required for the strand invasion step catalyzed by RAD51 nucleofilaments.

PARP14 has been previously identified as a transcriptional regulator (38). Indeed, a large part of the total PARP14 protein is found associated to chromatin (Figure 1). It is possible that its effect on recombination is in part mediated by this transcription regulation activity of PARP14. PARP14 was also identified as a negative regulator of NFκB pathway signaling initiated by extracellular signals (37). However, the NFκB pathway is also activated by DNA damage (54), and it is unclear if PARP14 can regulate this intracellular activation also. We investigated the transcription profile of PARP14-depleted HeLa cells using RNA-sequencing and observed no significant difference in the expression of known double strand break repair factors after DNA damage treatment (Supplementary Figure S15), arguing that transcription regulation is not the major mechanism by which PARP14 promotes recombination.

### PARP14 and replication stress

Replication of difficult endogenous templates such as CFS is prone to stalling and fork disassembly, resulting in chromosome breakage and translocations (14,15). Oncogene-induced hyper-proliferation in pre-cancerous cells was proposed to result in increased genomic instability particularly at CFS sites, since increased replication rates in these cells might not allow enough time to resolve replication forks stalled at these sites (16,17). Indeed, the FRA3B CFS is frequently found as site of translocations in cancer, resulting in the inactivation of the FHIT tumor suppressor gene that localizes at this site. DNA repair mechanisms such as HR and TLS were shown to protect against CFS breakage, presumably by promoting fork restart, and repair proteins such as 53BP1, ATR, RECQ1 have been found to be localized to CFS in response to replication inhibition by aphidicolin, an experimental method of accentuating fork stalling at CFS (47,48,51). Our original observation that PARP14 is associated with the replication machinery, and the known role of HR in alleviating replication stress at CFS and suppressing CFS breakage, prompted us to investigate if PARP14 also plays a role in CFS metabolism. Using ChIP experiments, we found that PARP14 localizes to the FRA3B and FRA7H CFS, suggesting that PARP14 not only participates in recombination reactions induced by DNA lesions, but also at endogenous difficult to replicate templates (Figure 5). Interestingly, RAD51 is required for resistance to aphidicolin and forms foci in response to aphidicolin treatment (52), suggesting that PARP14 and RAD51 functionally cooperate to promote replication of these sites.

In conclusion, we provide evidence that the mono-ADP-ribosyltransferase PARP14 is a novel modulator of HR playing an important role in double strand break repair, as well as in the replication of DNA lesions and of endogenous CFS. Our work suggests that PARP14 inhibitors can potentiate genotoxic chemotherapy by blocking HR DNA repair and rendering cancer cells hypersensitive to DNA damage.

## SUPPLEMENTARY DATA

Supplementary Data are available at NAR Online.

SUPPLEMENTARY DATA
