# Supplementary material for: A novel role for the mono-ADP-ribosyltransferase PARP14/ARTD8 in promoting homologous recombination and protecting against replication stress
Source: Nucleic Acids Res. 2015 Mar 9;43(6):3143–53. doi: 10.1093/nar/gkv147 (PMC4381061; doi:10.1093/nar/gkv147)
Supplement: SUPPLEMENTARY DATA [file supp_43_6_3143__index.html]

A novel role for the mono-ADP-ribosyltransferase PARP14/ARTD8 in promoting homologous recombination and protecting against replication stress — A novel role for the mono-ADP-ribosyltransferase PARP14/ARTD8 in promoting homologous recombination and protecting against replication stress — SUPPLEMENTARY DATA 

# A novel role for the mono-ADP-ribosyltransferase PARP14/ARTD8 in promoting homologous recombination and protecting against replication stress

## SUPPLEMENTARY DATA

**Files in this Data Supplement:**

- SUPPLEMENTARY DATA
