# Supplementary material for: A novel role for the mono-ADP-ribosyltransferase PARP14/ARTD8 in promoting homologous recombination and protecting against replication stress
Source: Nucleic Acids Res. 2015 Mar 9;43(6):3143–53. doi: 10.1093/nar/gkv147 (PMC4381061; doi:10.1093/nar/gkv147)

Legends to Supplementary Figures

Suppl. Fig. S1. PARP14 interacts with PCNA in co-immunoprecipitation experiments. A. Co-immunoprecipitation experiment showing that endogenous PCNA and PARP14 interact in HeLa cells. B. PCNA immunoprecipitation experiment from HeLa cells arrested in S-phase (by 2.5mM thymidine treatment for 18h, followed by recovery for two hours in drug-free media), or G2/M (by 100ng/ml nocodazole treatment for 18h). PARP14 co-precipitated with PCNA in S-phase, but not G2/M extracts. C. PCNA immunoprecipitation experiment from 8988T cells, showing that addition of DNA degrading enzyme benzonase (0.1U/μl) does not affect PARP14 binding. This indicates that the interaction is not mediated by DNA.

Suppl. Fig. S2. PARP14 interacts with PCNA in GST-pulldown experiments. A. Coomassie staining showing the recombinant GST-PCNA (and control GST-Empty) produced from bacteria and purified by GST affinity chromatography. B. GST-pulldown showing that PARP14 from HeLa cells extract interacts with GST-PCNA, but not with control GST-Empty. PCNA interaction with PAF is used as positive control.

Suppl. Fig. S3. PARP14 knockdown by siRNA. Western blot with anti-PARP14 antibodies showing that PARP14 protein levels are efficiently downregulated by siRNA in U2OS (A) and 8988T (B) cells. Asterisks indicate crossreactive bands.

Suppl. Fig. S4. γH2AX foci in PARP14-knockdown cells. A. γH2AX foci are not affected by PARP14 knockdown in the absence of DNA damage treatment in HeLa cells. The average of three independent experiments with at least 40 cells counted in each experiment is shown. Error bars represent standard deviations. B, C. γH2AX foci in U2OS cells are increased following exposure to HU (1mM for 12h) or UV (20J/m^2, analyzed 2h later). The average of four independent experiments with at least 40 cells counted in each experiment is shown. Error bars represent standard deviations.

Suppl. Fig. S5. Bi-dimensional BrdU/PI flow cytometry. A. Representative flow cytometry plots of HeLa cells, cycling or HU arrested/released as indicated, are shown. Arrows indicate the G2 population which is reduced in PARP14 knockdown cells after HU release. B, C. The percentage of mid-S-phase cells (R3: BrdU-positive, DNA content between 2N and 4N) and late-S-phase cells (R4: BrdU-positive, 4N DNA content) is shown (averaged from three independent experiments, -/+ standard deviations). P-values are also shown (ns=not significant).

Suppl. Fig. S6. Cell cycle distribution by PI staining, following release from HU arrest. Quantification showing the percent of HeLa cells in G2 under basal conditions, following incubation with HU (2mM for 18h), or 3h and 6h following removal of drug. Cell cycle distribution was analyzed by PI staining and flow cytometry. Bars represent the average of three independent experiments, and error bars are standard errors.

Suppl. Fig. S7. HU sensitivity upon PARP14 knockdown in 8988T cells. Cells were kept for three days in the presence of 0.4mM HU, and viability was assayed using CellTiterGlo reagent. Quantification of cellular viability from six independent experiments is shown. Error bars represent standard deviations.

Suppl. Fig. S8. UV-induced SupF mutagenesis assay in 293T cells, showing that PARP14 depletion does not affect TLS. The SupF plasmid was exposed to 1000J/m^2 UV dose, prior to transfection. The average of three independent experiments, with standard deviations as error bars, is shown.

Suppl. Fig. S9. Increased DNA breaks in PARP14-depleted U2OS and 8988T cells. Cells were exposed to UV (40J/m^2) and breaks were measured 4h later using the alkaline comet assay. The average comet tail length collected from two experiments (+/- standard errors), with at least 50 comets measured in each assay, is presented in the table (arbitrary units). The p-value calculated for all measurements in the two experiments is shown.

Suppl. Fig. S10. DNA fiber assay showing reduced replication tract length in PARP14 knockdown cells. U2OS (A) and 8988T (B) cells were treated with 1μM CPT. At least 50 fibers for each condition were measured and included in the analysis. Error bars represent standard errors.

Suppl. Fig. S11. RAD51 foci in PARP14-depleted U2OS cells. Shown is the percentage of cells with more than five RAD51 foci, analyzed 4h after recovery from 1μM CPT treatment. The average of three independent experiments, with standard errors as error bars, is shown.

Suppl. Fig. S12. Bleomycin sensitivity upon PARP14 knockdown in 8988T cells. Cells were kept for three days in the presence of 30μM Bleomycin, and viability was assayed using CellTiterGlo reagent. Quantification of cellular viability from four independent experiments is shown. Error bars represent standard deviations.

Suppl. Fig. S13. RAD51-PARP14 interaction. A. GST-Macro2 pulldown with extracts of control or PARP14-knockdown UV-treated HeLa cells (100J/m^2 analyzed 2h after exposure). PARP14 depletion reduces binding of GST-Macro2 to RAD51. B. Similar experiment using extracts of U2OS cells. Two different PARP14 oligonucleotides result in similar reduction in interaction, showing that it is not due to off target effects. C. Quantification of the RAD51-Macro2 interaction in control and PARP14-depleted cells. ImageJ was used to quantify RAD51 band intensities from GST-Macro2-pulldowns. The average of three independent experiments, with standard deviations, is shown. D. RAD51 immunoprecipitation from UV-treated 8988T cells (100J/m^2 analyzed 2h after exposure) showing co-precipitation of endogenous PARP14.

Suppl. Fig. S14. Aphidicolin sensitivity upon PARP14 knockdown in 8988T cells. Cell survival assay showing that PARP14 knockdown in 8988T cells results in aphidicolin sensitivity. Cells were incubated for three days in the presence of 10μM aphidicolin, and viability was measured using the CellTiterGlo reagent. The average of four independent experiments, with standard deviations as error bars, is shown.

Suppl. Fig. S15. RNA-sequencing data showing that PARP14 knockdown does not affect the mRNA expression of double strand break repair genes. HeLa cells treated with 1μM CPT for 2h and harvested 4h later, were subjected to mRNA extraction followed by next generation sequencing. The expression of 42 genes known to participate in double strand break repair, from two independent experiments, is shown. The ratio of the expression of each gene in siPARP14 cells to its expression in siControl cells is shown. In the first bar, the expression of PARP14 itself is presented, showing the reduction in expression caused by siRNA. All double strand break repair genes investigated show no significant changes in expression, having fold change values close to 1.

Supplementary Figure S1.

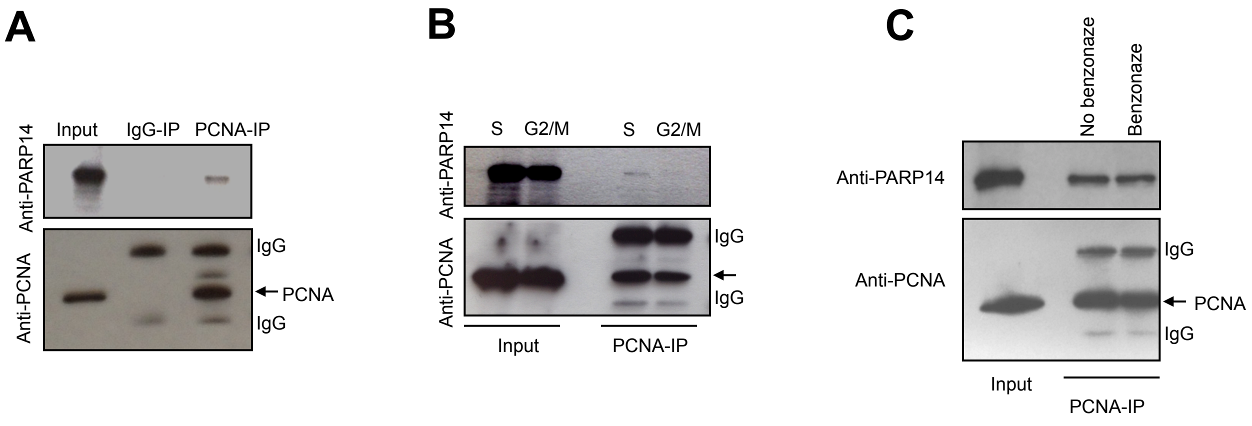

Supplementary Figure S2.

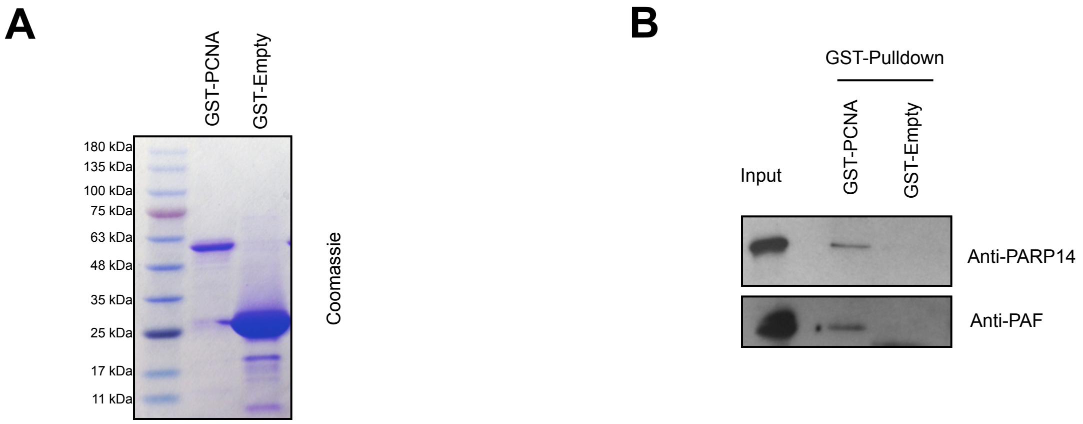

Supplementary Figure S3.

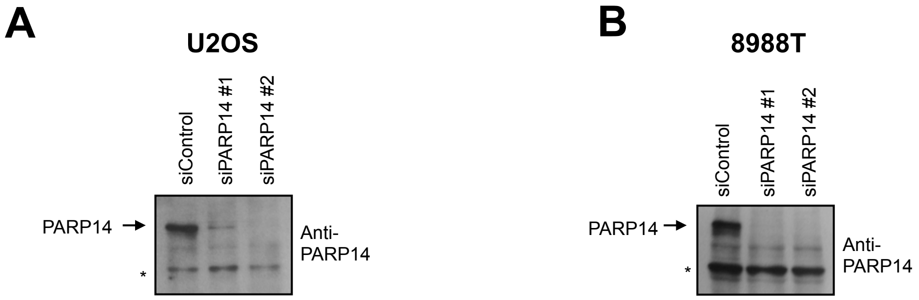

Supplementary Figure S4.

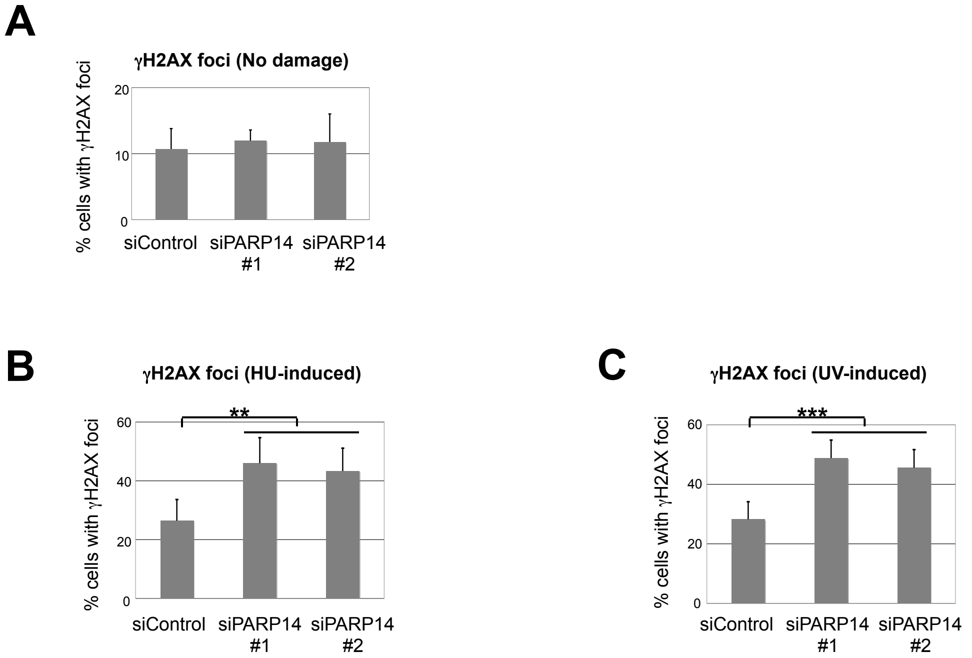

Supplementary Figure S5.

A

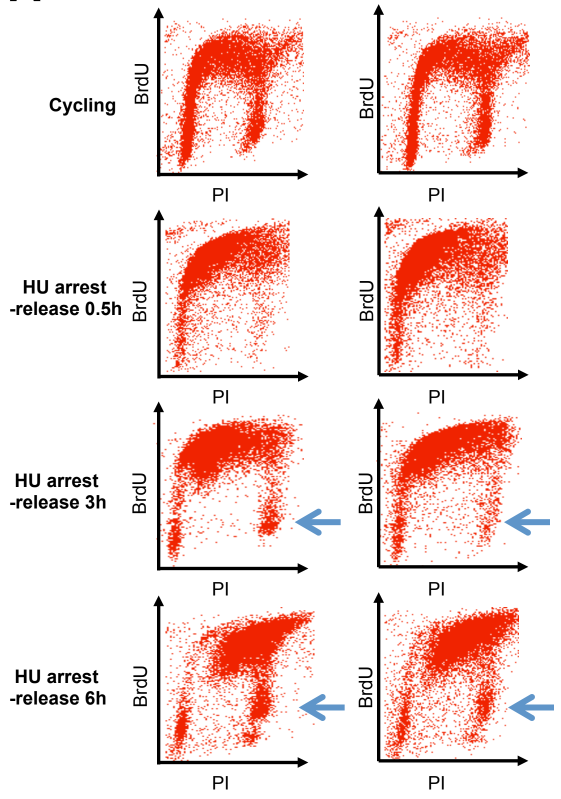

B

Mid-S-phase (R3)

	siControl	siPARP14	
Cycling	19 +/-4	19 +/-4	ns
HU 0.5h	21 +/-2	25 +/-2	ns
HU 3h	43 +/-2	35 +/-3	**p<0.05**
HU 6h	49 +/-2	55 +/-4	ns

C

Late-S-phase (R4)

	siControl	siPARP14	
Cycling	13 +/-2	16 +/-4	ns
HU 0.5h	6 +/-2	7 +/-2	ns
HU 3h	11 +/-1	6 +/-1	**p<0.05**
HU 6h	27 +/-2	18 +/-3	**p<0.05**

Supplementary Figure S6.

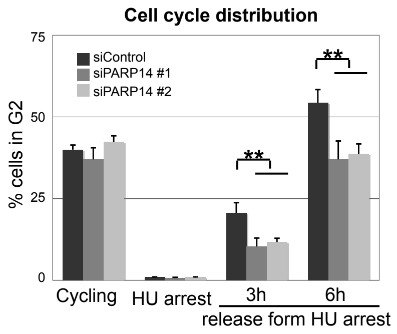

Supplementary Figure S7.

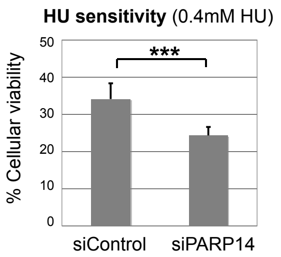

8988T

Supplementary Figure S8.

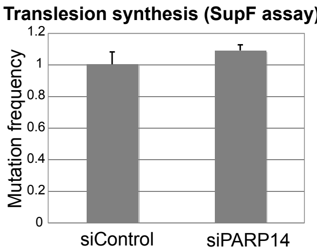

Supplementary Figure S9.

	siControl	siPARP14	
U2OS	106 +/-4	128 +/-3	$p<10^{-6}$
8988T	123 +/-2	145 +/-3	$p<10^{-13}$

Supplementary Figure S10.

A

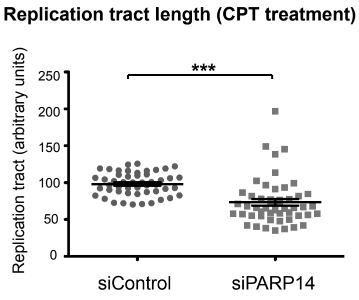

U2OS

B

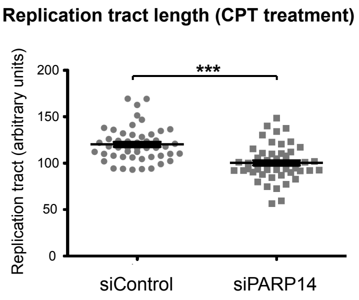

8988T

Supplementary Figure S11.

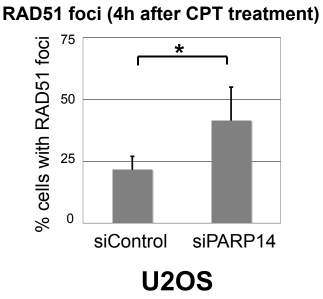

Supplementary Figure S12.

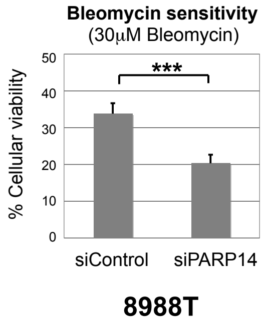

Supplementary Figure S13.

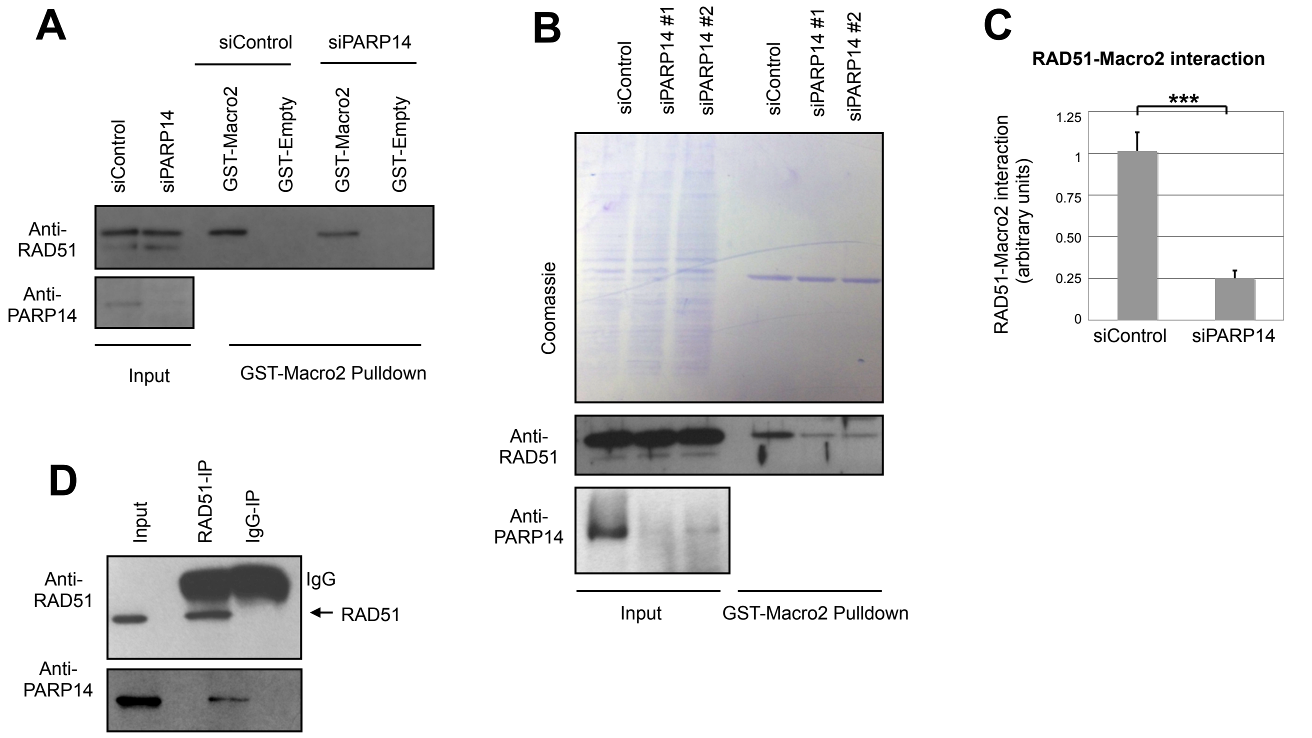

Supplementary Figure S14.

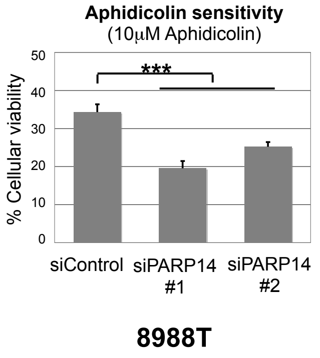

Supplementary Figure S15.

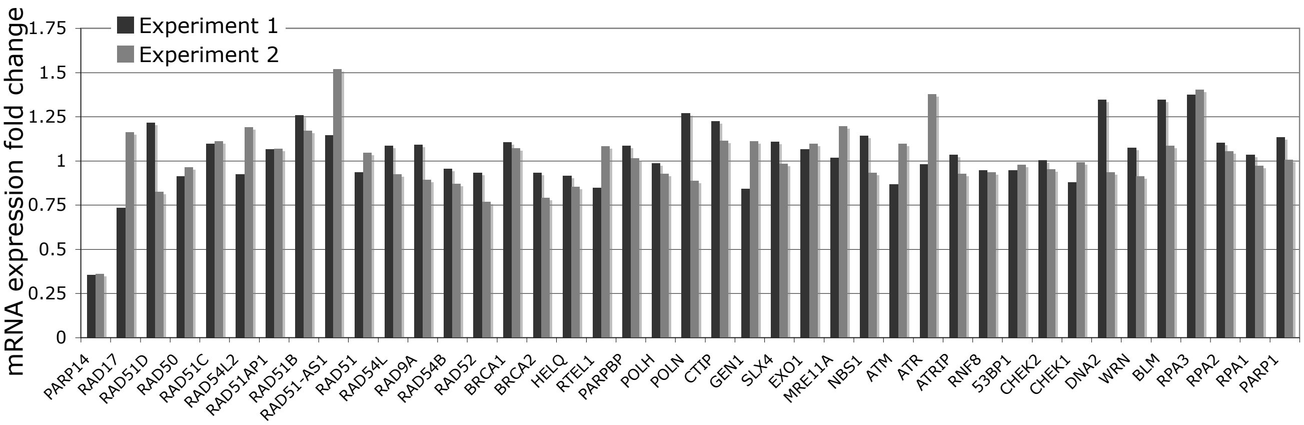

Supplement: SUPPLEMENTARY DATA [file supp_gkv147_nar-03367-d-2014-File007.pdf]
